# A two-stage deep learning framework for lead instrument recognition in polyphonic music featuring Chinese instruments

**DOI:** 10.1371/journal.pone.0327442

**Published:** 2026-04-29

**Authors:** Jiaxiang Zheng, Moxi Cao, Chongbin Zhang

**Affiliations:** 1 Department of Global Cultural Convergence, Graduate School, Kangwon National University, Chuncheon-si, Gangwon-do, South Korea; 2 School of Modern Music and Technology, Nanjing University of the Arts, Nanjing, Jiangsu, China; University of Kurdistan Hewler, IRAQ

## Abstract

Rapid advancement of deep learning methodologies has considerably progressed the domain of automatic music analysis; however, several challenges remain in adapting these approaches to the intricate and multifaceted realm of Chinese traditional music. In particular, the task of discerning lead instruments within polyphonic textures, a fundamental element of Chinese ensemble performance, has not been sufficiently explored, and current models frequently fail to address the complex acoustic features and structural nuances present in real-world recordings. To bridge this gap, we propose a deep learning framework for Chinese instrument recognition employing a two-stage “separation-then-classification” strategy. Initially, a source separation module is deployed to extract individual instrument representations from mixed audio, which is subsequently followed by a multi-label classification network to identify the target instruments. Empirical results from a newly constructed dataset reveal marked enhancements in accuracy, precision, recall, and F1 score metrics, with especially notable improvements in distinguishing instruments similar in timbral characteristics, such as dizi and xiao. This study offers a novel approach to the recognition of polyphonic instruments within traditional music contexts, presenting a scalable and model-agnostic improvement strategy. It also laid the methodological and technological foundations for the preservation, analysis, and promotion of the cultural heritage of Chinese music through intelligent audio systems. The code is available at: https://github.com/CB389636/chic.

## Introduction

The field of music information processing has experienced notable advancements due to the progression of artificial intelligence and computational audio techniques. The automatic recognition of musical instruments is a fundamental task within this domain and plays a crucial role in applications including music retrieval, intelligent education, and performance analysis. Furthermore, it facilitates critical capabilities for the comprehension and creation of structured music. Although extensive research has yielded comprehensive methodologies ranging from audio feature extraction to multilabel classification for standard Western instruments, the modeling of Chinese traditional instruments—characterized by their rich cultural significance and complex expressive forms—remains in an exploratory phase and lacks systematic solutions.

Chinese traditional music is characterized by a significant level of complexity and lack of standardization with respect to timbral structure, modal systems, and performance techniques. In contrast to the relatively standardized instrumentation found in Western orchestral music, Chinese instrumental music is frequently organized into diverse ensemble formats with multi-layered structures, encompassing lead, accompaniment, percussion, and ornamental components, thereby exhibiting distinctive polyphonic textures. While earlier studies have begun to advance the classification of instrument timbre, employing convolutional neural networks and time-frequency feature fusion to discern individual traditional instruments, there remain several critical limitations in contemporary mainstream research.

Primarily, a significant number of studies concentrate on idealized solo or unaccompanied contexts, thereby omitting models capable of encapsulating the complex interrelations among multiple components within genuine ensemble settings. Additionally, datasets that are synthetic or manually segmented are often utilized, failing to represent the inherent polyphonic complexity associated with traditional music in its natural performance environment. Furthermore, classification labels often remain rudimentary, limited to ’instrument names’, without advancing towards a hierarchical semantic labeling system that facilitates structural comprehension. Moreover, the majority of extant algorithms have been developed with corpora sourced from popular or Western classical music, and their applicability to Chinese traditional music remains to be established. Finally, real-world application scenarios, such as the documentation of national music performances, intelligent pedagogical feedback, and virtual performance systems, predominantly involve polyphonic tracks with accompaniment. In these practical scenarios, current models typically encounter performance constraints and demonstrate limited capacity for generalization.

In light of the challenges previously discussed, the creation of a traditional Chinese instrument recognition system capable of functioning efficiently within real-world polyphonic contexts with the ability to comprehend multipart interactions has emerged as an exigent task in this domain. Traditional monophonic recognition models prove inadequate in addressing complexities such as instrument masking, spectral overlap, and role blending during ensemble performances. Consequently, enhancements in modeling strategies, data structures, and labeling frameworks are necessary.

In response to this issue, the current study introduces a recognition system specifically oriented towards polyphony for Chinese traditional instruments, employing a two-stage source separation classification framework to adeptly address the complexities arising from multi-part structures. In particular, we have developed a high-caliber dataset of Chinese traditional music, consisting of 1,612 segments of real-world performances with durations ranging from 10 to 60 seconds. This dataset encompasses four prevalent Chinese instruments—guzheng, pipa, xiao, and dizi—and includes accompaniment tracks and varied ensemble configurations, thereby providing significant polyphonic diversity and extensive timbral coverage. This dataset is designed to facilitate multiple tasks, such as large-scale polyphonic instrument classification, part recognition, and structural alignment.

Utilizing this dataset as a foundation, we develop a role-sensitive labeling framework that annotates each audio track based on its functional role within the ensemble—classified as lead, accompaniment, or ornamental. This approach enables the model to discern not only ’which instrument is playing,’ but also ’the function it serves within the ensemble.’ In addition, we investigate the adaptability of various source separation models within the context of Chinese traditional music and integrate these models with deep neural networks to establish a multilabel classification system. This system is designed to accurately recognize multiple instruments from real-world polyphonic audio inputs.

This study introduces novel approaches and methodologies for the large-scale automatic recognition of traditional Chinese musical instruments across three principal dimensions: data construction, system design, and label modeling. The proposed framework addresses a notable gap in the application of source separation technologies within the domain of traditional Chinese music. Furthermore, it establishes foundational support for multipart structure modeling, multilabel classification, and the comprehension of semantic structures. The outcomes of this research find extensive application in diverse contexts, including music education, the digital preservation of intangible cultural heritage, intelligent retrieval of traditional music, and virtual orchestration systems, thereby underscoring both its significant academic value and its potential for cultural dissemination.

## Related work

### Instrument classification methods

The study of automatic instrument classification has a long-established history within the domain of music information retrieval. Herrera et al. [1] have comprehensively classified extant methodologies into two principal categories: unsupervised approaches that prioritize the perception of timbral similarity and supervised recognition methods grounded in predefined instrumental categories. Initial investigations predominantly depended on manually crafted audio features, integrated with conventional machine learning classifiers, to identify monophonic instrument signals [2–4]. For instance, Eronen et al. [2] employed frequency domain features, such as Mel-Frequency Cepstral Coefficients (MFCCs), and trained Hidden Markov Models (HMMs) to classify the tones of orchestral instruments. Agostini et al. [3] executed spectral analysis to extract timbral characteristics and trained classifiers to attain superior recognition accuracy. Livshin et al. [4] amalgamated various audio features and applied feature selection techniques to boost the generalization performance across datasets.

Furthermore, numerous investigations have conducted comparative analyses of various classification algorithms. Marques and Moreno [5] assessed the efficacy of Gaussian Mixture Models (GMMs) and Support Vector Machines (SVMs) within the realm of instrument classification tasks. To facilitate standardized evaluation benchmarks, multiple internationally accessible datasets have been made available. For instance, the RWC Musical Instrument Sound Database from Japan provides high-quality scale-tone recordings encompassing a wide array of instruments, thereby supporting uniform algorithmic comparison [6].

With advancements in this domain, scholars have proposed several enhancements in methodologies for feature extraction and classification. Deng et al. [7] conducted a systematic analysis of the discriminative capabilities of different audio features, identifying key combinations that effectively differentiate instruments. Essid et al. [8] devised a pairwise voting fusion strategy for binary classifiers, which markedly enhanced the accuracy of classifying solo instrument recordings. Benetos et al. [9] employed nonnegative matrix factorization (NMF) alongside feature subset selection to model timbral characteristics and facilitate instrument classification. Eronen et al. [10] achieved further improvements in recognition performance by incorporating Independent Component Analysis (ICA) transformations into feature sets and training discriminative hidden Markov models (HMMs). Motivated by biological neural frameworks, Newton and Smith [11] developed a model based on Echo State Networks (ESN), leveraging transient attack segments of sounds to enhance classification accuracy.

With the evolution of instrument classification from monophonic to polyphonic music contexts, researchers have begun to tackle the complexities associated with the recognition of multiple concurrently active instruments. Kitahara et al. [10,12] introduced feature weighting alongside pitch-related modeling methodologies aimed at alleviating the impact of sound overlaps resulting from the simultaneous performance of instruments. Meanwhile, Eggink and Brown [11,13] implemented a missing feature approach, disregarding occluded spectral components in composite audio signals to enhance the efficacy of instrument identification.

Subsequent investigations employed source separation methodologies to enhance classification efficacy in polyphonic contexts. For instance, Heittola et al. [14] introduced a pre-separation technique predicated on a source–filter model prior to recognition, facilitating the extraction of features specific to individual instruments. Duan et al. [15] developed novel cepstral features aimed at characterizing the timbral characteristics within multipitch mixtures. Bosch et al. [16] performed a comparative analysis of various sound separation algorithms and assessed their influence on the recognition of primary instruments. Furthermore, Fuhrmann et al. [17] examined the scalability and generalizability of instrument recognition systems in the context of complex musical signals.

In recent years, significant advancements have been achieved in the domain of instrument classification through the application of deep learning techniques. Lee et al. [18] utilized deep belief networks (DBNs) for the unsupervised learning of audio features, facilitating instrument classification without reliance on manually crafted features. Building on this foundation, Dieleman and Schrauwen [19] introduced an end-to-end Convolutional Neural Network (CNN) architecture capable of learning discriminative representations directly from raw audio waveforms. Han et al. [20] employed deep CNNs to discern dominant instruments within multi-instrument mixtures, thereby accomplishing notable enhancements in classification performance within complex acoustic environments. Additionally, Choi et al. [21] utilized fully convolutional networks for the task of automatic music tagging, enabling the concurrent recognition of multiple instrument categories within an audio segment.

Supported by the availability of open large-scale datasets, recent investigations have broadened both the scope and the intricacy of instrument classification. For instance, Blaszke et al. [22] have evidenced that models based on deep learning are capable of effectively identifying multiple co-occurring instruments within audio segments. The dissemination of the OpenMIC-2018 dataset [23] has furnished extensive annotated data, facilitating the training of multi-instrument recognition models. Furthermore, numerous enhancements have been introduced to address specific challenges. These include advances in feature representations such as refined group delay features [24] and sparse cepstral coefficients [25], recognition of instruments at the musical phrase level [26], and analyses concentrating on the classification of solo instrument recordings [2].

Concerning the interpretability of the model, Chen et al. [27] undertook initial investigations employing visualization techniques to scrutinize the impact of various spectrogram representations on CNN-based decisions, thereby providing insightful contributions towards the comprehension of deep learning models in the context of instrument recognition tasks.

Despite the considerable advancements achieved in the domain of instrument classification, particularly the efficacy of deep learning in automating feature extraction, bolstering robustness in polyphonic contexts, and enhancing the generalization ability of models, extant systems continue to encounter numerous unresolved issues. Firstly, the challenge of feature entanglement, prompted by the simultaneous presence of multiple instruments within polyphonic music, remains a primary impediment. Numerous end-to-end models experience difficulties in accurately disentangling lead and accompaniment instruments, often adversely affected by extraneous background noises or environmental disturbances [13,14,17]. Secondly, while certain studies have endeavored to integrate source separation mechanisms, these processes are generally treated in isolation rather than through a jointly optimized approach. This disjointed strategy results in the propagation of errors from the separation process into the classification stage [15,16]. Thirdly, the majority of existing research remains predominantly concentrated on Western orchestral instruments, thereby lacking systematic approaches for modeling the timbral characteristics and performance contexts of traditional Chinese instruments. This deficiency undermines the adaptability of current methodologies in multicultural music scenarios [6,22].

### Research on classification of Chinese traditional instruments

In recent years, the field of audio analysis for traditional Chinese and other ethnic instruments has experienced substantial advances. In relation to the task cited in instrument category classification, several comprehensive datasets have been formulated to facilitate research efforts. The Chinese Traditional Instrument Sound Library (CTIS) encompasses recordings from 287 traditional and ethnic minority Chinese instruments, making it the most extensive public dataset available to date. Nonetheless, systematic evaluation has been conducted on only approximately one-third of these instruments, encompassing 78 categories [28]. In response to the inadequacy of publicly accessible data, Wei et al. [29] introduced the ChMusic dataset, which comprises 55 solo audio recordings covering 11 traditionally encountered Chinese instruments, thus instituting a standardized evaluation protocol. Using such data sets, deep learning-based classification models have demonstrated remarkable efficacy; in particular, convolutional neural networks that use Mel-spectrogram input have achieved classification accuracies that surpass 99% for categories of Chinese instruments [30].

Yet, the endeavor of lead instrument recognition, the identification of the primary melodic instrument within multi-instrumental ensembles, remains significantly underexplored. Most of the studies in existence concentrate on solo recordings and do not possess dedicated datasets or algorithmic frameworks capable of automatically discerning lead instruments within the polyphonic contexts of traditional Chinese music. This deficiency signifies a substantial challenge for the domain.

In addition, numerous studies have investigated the automatic transcription and symbolic representation of traditional Chinese instrumental performances, thus contributing to the field of instrument classification research. For example, the GQ39 dataset, developed by Huang et al. [31], offers note-level annotations for 39 solo guqin compositions, facilitating the examination of performance techniques and pitch patterns. Furthermore, Wang et al. [32] introduced PipaSet, the premier multimodal dataset for automatic transcription of pipa music, including audio recordings, notated scores and multicamera video footage. This dataset allows the extraction of pitch, duration of onset, and detailed playing techniques in solo pipa performances. Additionally, institutions such as the Central Conservatory of Music have released the CCOM-HuQin dataset, which encompasses 11,992 segments of the bow technique and 57 annotated compositions for instruments belonging to the huqin family. This collection helps conduct research focused on the recognition of performance techniques within Chinese bowed string instruments [33].

On the algorithmic front, both traditional machine learning and deep neural networks have been applied to the classification of traditional Chinese instruments. For example, Liu [34] used a Softmax regression model combined with a backpropagation (BP) neural network to automatically recognize 25 pitch classes of the ruan (a plucked lute in the pipa family), achieving an average accuracy of 95.6%.

In summary, existing research on audio analysis of Chinese traditional instruments has achieved promising results in instrument category classification tasks, particularly under monophonic conditions, where high recognition accuracies have been reported. However, real-world musical scenarios more frequently exhibit composite structures involving a lead instrument accompanied by one or more supporting instruments. Such ensemble settings introduce significant interference when attempting to identify the lead instrument directly, as the acoustic overlap and timbral blending between the melody and accompaniment parts substantially degrade classification performance. However, most publicly available datasets consist primarily of solo or isolated recordings, and current methods are predominantly focused on single-instrument classification, making them insufficient for practical applications.

In order to address this identified gap, we propose an automated recognition framework specifically tailored for the recognition of Chinese traditional instruments within polyphonic and multisource musical environments. This framework employs a two-stage strategy comprising “separation followed by classification,” with the objective of precisely identifying the lead instrument in polyphonic performances of Chinese music. Initially, a deep learning-based source separation model undergoes pre-training to extract relatively independent source components from complex mixed audio, circumventing the dependence on manual annotations. Subsequently, the extracted audio features are transmitted to an instrument classification model for recognition purposes. This methodology effectively reduces interference from accompanying instruments and enhances the accuracy of identifying the lead instrument.

The proposed strategy proficiently mitigates the fundamental issue of “feature entanglement,” which is intrinsic to conventional methodologies, thereby broadening the applicability of instrument recognition systems to encompass non-Western musical paradigms, including Chinese traditional music. Furthermore, it illustrates the theoretical robustness and practical efficacy of simultaneously modeling source separation and classification within the realm of instrument recognition endeavors.

## Materials and methods

### System overview

This research focuses on the challenge of automatically recognizing traditional Chinese musical instruments in recordings with multiple sources by introducing a dual-stage deep learning architecture grounded in a ’separation-then-classification’ methodology. The system processes polyphonic recordings with multiple instruments as input, employs a source separation network to isolate distinct features for each target instrument, and subsequently utilizes a classification network to conduct multilabel predictions concerning the presence of each instrument. This methodology allows for precise identification of several traditional Chinese instruments within complex audio signals.

The overall architecture is composed of two functional components. The front-end, referred to as the Separation Network, transforms the input multi-instrument mixture into several high-dimensional feature representations, each corresponding to a distinct instrument class. The back-end, known as the Classification Network, is responsible for modeling and deriving decisions from the extracted features, thereby generating confidence scores for each instrument. This modular configuration effectively separates signal interference caused by overlapping sources, converting the traditional end-to-end classification task into a structured process consisting of ’representation extraction’ and ’decision inference.’

Initially, the mixed audio input undergoes transformation into a time-frequency representation through the utilization of the Short-Time Fourier Transform (STFT). This representation is subsequently processed by the separation network, which executes frequency band partitioning and discriminative encoding. The network employs attention mechanisms alongside recurrent neural networks to comprehensively model time-frequency features, ultimately generating independent feature tensors for each target instrument. In comparison to direct classification methods that rely on mixture signals, this approach facilitates a more accurate concentration on the spectral and temporal attributes of individual instruments, thereby improving the modeling of fine-grained timbral and performance features.

The classification network engages in multilabel learning predicated upon the extracted feature vectors. Considering that Chinese traditional instruments frequently manifest in ensemble contexts, the model undergoes training with multi-label supervision and utilizes sigmoid activation to independently ascertain the presence of each instrument, thereby facilitating co-occurrence modeling. Additionally, to enhance robustness against class imbalance and overlapping categories, the classification module integrates label balancing strategies and employs weighted loss functions, thus optimizing recognition performance in authentic performance environments.

The proposed separation-then-classification architecture notably enhances the system’s capacity to identify complex overlapping instrumental signals. Additionally, it establishes a robust and modular basis for subsequent tasks, including performance style analysis and automatic orchestration modeling.

### Instrument separation network

**Fig 1:**
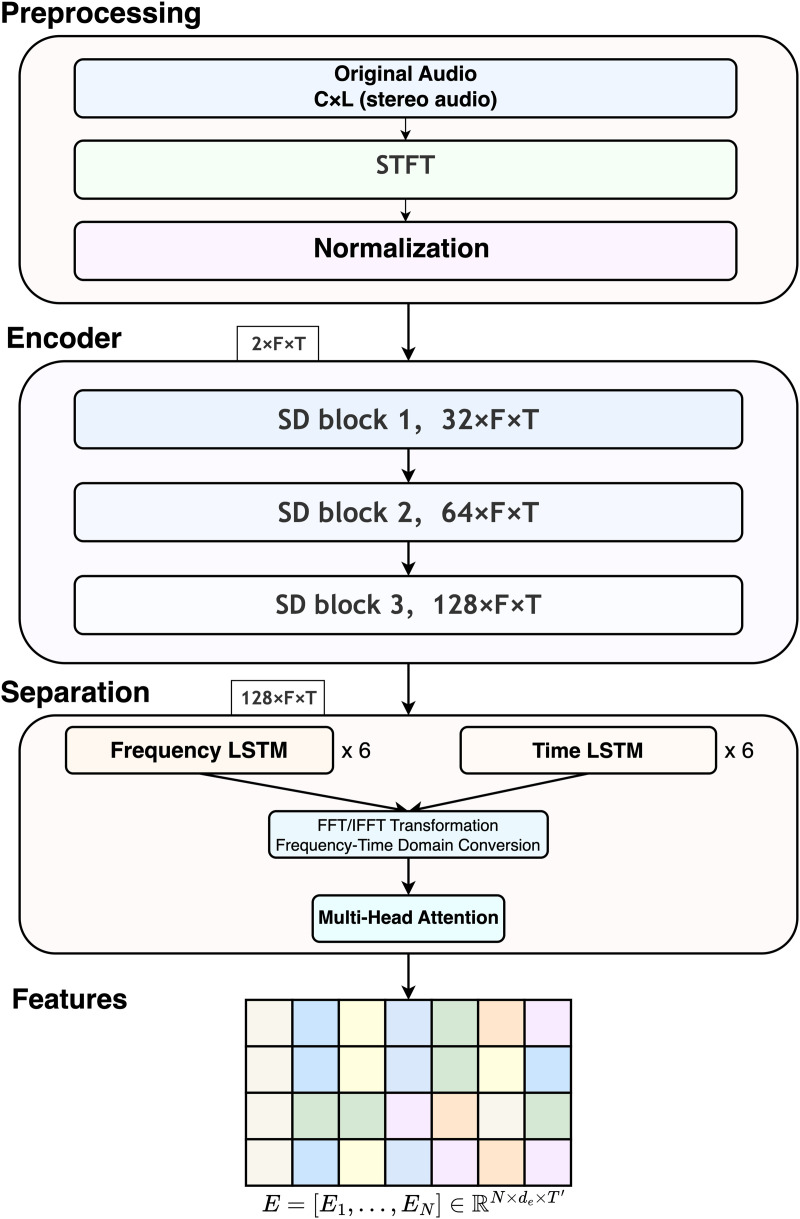
Architecture of the source separation network. The network consists of preprocessing, encoding with SD (Spectral Domain) blocks, and dual-domain separation using LSTM and multi-head attention. The encoder input dimension is 2 × *F* × *T*, where the factor of 2 corresponds to the two channels of stereo audio input. The output is a feature tensor for instrument classification.

The aim of the separation network is to systematically model mixed-audio inputs comprising multiple instruments and to derive multichannel representations that encapsulate the distinct features of each traditional Chinese instrument. These representations provide input that is both untainted and discriminative for subsequent classification tasks. The network is constructed on a frequency band-specific processing framework, incorporating convolutional, recurrent, and attention mechanisms to thoroughly capture the spectral distribution, harmonic structure, and temporal dynamics intrinsic to Chinese instruments. The overall architecture of the separation network is illustrated in Fig 1.

#### Time-frequency preprocessing.

Prior to being processed by deep neural networks, raw audio signals represented in one-dimensional time-domain must undergo transformation into time-frequency representations. This transformation facilitates the model’s ability to capture timbral characteristics and harmonic structures across the frequency dimension. In the present study, the Short-Time Fourier Transform (STFT) is employed to convert the input signal into a joint time-frequency domain representation, thereby enabling the model to leverage both local temporal and spectral information. The input audio is processed in stereo format, preserving the two-channel structure throughout the preprocessing stage. For each channel, let the input mixture signal be denoted by $x\in {\mathbb{R}}^{L}$, where *L* represents the total number of samples at a sampling rate *f*_*s*_. The application of STFT produces the complex-valued time-frequency spectrogram *X*:


$$X=\text{STFT}(x)\in {\mathbb{C}}^{F\times T}$$ (1)

In this context, $F=\frac{n_{\text{fft}}}{2}+1$ refers to the frequency resolution, defined as the number of frequency bins per frame, while $T=\left\lfloor \frac{L+p-n_{\text{fft}}}{n_{\text{hop}}}\right\rfloor +1$ signifies the count of time frames. Parameter *n*_fft_ indicates the length of the Fourier window, *n*_hop_ denotes the hop size between successive frames, and *p* pertains to the zero padding length applied to the entire signal before framing. This transformation proficiently divides the time-domain signal into overlapping windows, subsequently applying a local Fourier transform to each window in order to capture the temporal dynamics of the spectral structure.

In our implementation, we configure *n*_fft_ = 1024 to achieve an optimal balance between frequency resolution and computational efficiency. A hop size of $n_{\text{hop}}=\frac{1}{4}n_{\text{fft}}=256$ is selected to ensure adequate overlap, thereby enhancing temporal continuity and increasing the robustness of the short-time spectra. The window function plays a crucial role in the spectral representation; accordingly, we employ the Hann window to achieve an equilibrium between mainlobe width and sidelobe attenuation, effectively mitigating spectral leakage.

The complex-valued spectrogram *X* is subsequently decomposed into its magnitude and phase components. The magnitude spectrogram $A=|X|\in {\mathbb{R}}^{F\times T}$ represents the distribution of energy across time and frequency, and it serves as the fundamental feature for modeling timbre, formants, and harmonic structures. The phase component ∠X, primarily utilized for waveform reconstruction, is not considered in the modeling processes undertaken in this study. Therefore, within the feature extraction pipeline, only the magnitude spectrogram *A* is retained as input for the downstream models, while the phase information is discarded. Since the input audio is in stereo format, the magnitude spectrograms from both left and right channels are stacked along the channel dimension, resulting in a combined input tensor of dimension 2 × *F* × *T* for the subsequent encoder, where the factor of 2 represents the two stereo channels.


$$A=|X|\in {\mathbb{R}}^{F\times T}$$ (2)

This time-frequency representation not only maintains the spectral characteristics specific to each instrument but also augments the model’s capability to disentangle heterogeneous signals. Notably, the frequency resolution afforded by the Short-Time Fourier Transform (STFT) establishes a robust foundation for ensuing frequency band modeling and time-frequency attention mechanisms, which are critical in the separation of overlapping traditional Chinese instruments.

#### Multi-band decomposition and encoding.

Inspired by SCNet [35], a frequency-domain source separation model that explicitly splits the mixture spectrogram into subbands and applies differentiated compression based on information density, this study introduces a multiband decomposition strategy to address the non-uniform spectral characteristics of Chinese traditional instruments, particularly the regular concentration of energy in low- to mid-frequency ranges. The overall magnitude spectrogram $A\in {\mathbb{R}}^{F\times T}$ is partitioned along the frequency axis into three subbands, each designed to capture different types of spectral content, such as fundamental frequencies, harmonic groups, and high-frequency residuals:


$$A^{\left(1\right)}=A[1:k_{1},:],\;A^{\left(2\right)}=A[k_{1}:k_{2},:],\;A^{\left(3\right)}=A[k_{2}:F,:]$$ (3)

The band boundaries *k*_1_ and *k*_2_ are determined based on predefined proportional parameters $\alpha_{1}$, $\alpha_{2}$, and $\alpha_{3}$, satisfying:


$$k_{1}=\lceil F\cdot \alpha_{1}\rceil ,\;k_{2}=\lceil F\cdot (\alpha_{1}+\alpha_{2})\rceil ,\;\alpha_{1}+\alpha_{2}+\alpha_{3}=1$$ (4)

The core idea of this band decomposition mechanism is to enable differentiated modeling of frequency information: the low-frequency band retains higher resolution to capture the detailed structure of fundamentals; the mid-frequency band focuses on the arrangement of overtones and harmonic density; and the high-frequency band, which mainly carries brightness and percussive components, is processed at lower resolution to reduce computational cost.

In order to ascertain the optimal proportions of the band, a series of ablation experiments was conducted to assess the effects of diverse α configurations on the accuracy of subsequent classification tasks. From several prospective configurations, the final arrangement was identified as $\alpha_{1}=0.2375$, $\alpha_{2}=0.3624$, and $\alpha_{3}=0.4001$, which correspond to frequency partitions of 23.75%, 36.24%, and 40.01%, respectively. This configuration consistently resulted in enhanced classification performance across various model architectures and dataset scenarios, thereby demonstrating a balanced compromise between maintaining low-frequency structural integrity and compressing high-frequency dimensions.

During the encoding stage, each subband *A*^(i)^ is fed into a 2D convolutional encoder module with a shared structure. Each encoder comprises a sequence of 2D convolutional layers (Conv2D), batch normalization (BatchNorm), and ReLU activation, forming a standard feature extraction pipeline:


$$Z^{\left(i\right)}=\text{Conv2D}(A^{\left(i\right)})\in {\mathbb{R}}^{C_{i}\times T^{\prime }}$$ (5)

Here, *C*_*i*_ denotes the number of output channels for band *i*, and *T*′ represents the number of time frames after temporal downsampling. This stage aims to reduce the dimensionality of the original time-frequency representation and to extract local structures and spectral patterns from each band, providing a comparable embedding space for unified downstream modeling.

The integration of a multiband decomposition strategy with convolutional encoders markedly enhances the model’s capability to capture subtle timbral variations across diverse instruments, while concurrently diminishing the overall computational complexity. This architecture is especially beneficial for the processing of multisource signals from Chinese traditional instruments.

#### Time-frequency modeling and attention mechanism.

In order to augment the model’s ability to capture both temporal and spectral dependencies within audio signals, a unified time-frequency fusion mechanism is introduced subsequent to the frequency-band encoding phase. This mechanism incorporates a bidirectional long-short-term memory (BiLSTM) network in conjunction with a multihead attention module (MHA) to concurrently model local performance dynamics and global cross-band structures, thereby facilitating more enriched representations of the expressive variations inherent in Chinese traditional instruments.

Specifically, the encoded convolutional features *Z*^(i)^ from each frequency band are first projected into a unified embedding space via a linear transformation to facilitate subsequent fusion and temporal modeling:


$${\tilde{Z}}^{\left(i\right)}=\text{Linear}(Z^{\left(i\right)})\in {\mathbb{R}}^{d\times T^{\prime }}$$ (6)

These bandwise embeddings are then concatenated along the channel dimension to form a joint representation:


$$\tilde{Z}=[{\tilde{Z}}^{\left(1\right)};{\tilde{Z}}^{\left(2\right)};{\tilde{Z}}^{\left(3\right)}]\in {\mathbb{R}}^{d^{\prime }\times T^{\prime }},\;d^{\prime }=\sum_{i}d$$ (7)

To capture contextual information in different time steps, we apply a BiLSTM network to $\tilde{Z}$. The bidirectional structure allows the model to incorporate both forward and backward sequential dependencies, improving its ability to represent performance-related temporal dynamics:


$$H=\text{BiLSTM}(\tilde{Z})\in {\mathbb{R}}^{d^{\prime \prime }\times T^{\prime }}$$ (8)

Building upon this foundation, we integrate a Multi-Head Attention mechanism to enhance the model’s capacity for discerning interfrequency relationships. By mapping feature representations into multiple subspaces and conducting parallel attention operations, the model is able to identify global frequency domain dependencies from a variety of perspectives. The ultimate attention output is calculated as follows:


$$H^{\prime }=\text{MHA}(H)=\text{Concat}({\text{head}}_{1},\ldots ,{\text{head}}_{h})W^{O}\in {\mathbb{R}}^{d^{\prime \prime }\times T^{\prime }}$$ (9)

Each attention head is computed as:


$$\begin{array}{cc} {\text{head}}_{j} & =\text{Softmax}\left(\frac{Q_{j}K_{j}^{⊤}}{\sqrt{d_{k}}}\right)V_{j},\\ Q_{j} & =HW_{j}^{Q},\;K_{j}=HW_{j}^{K},\;V_{j}=HW_{j}^{V} \end{array}$$ (10)

where $W_{j}^{Q},W_{j}^{K},W_{j}^{V}\in {\mathbb{R}}^{d^{\prime \prime }\times d_{k}}$ are the projection matrices for the query, key and value representations, and $W^{O}\in {\mathbb{R}}^{hd_{k}\times d^{\prime \prime }}$ is the output projection matrix to combine the attention heads.

The integration of BiLSTM and Multi-Head Attention in this research is prompted by two principal considerations. Firstly, LSTM networks are particularly adept at capturing sequential dynamics between consecutive temporal segments, which are prevalent in musical signals. Secondly, attention mechanisms address the inherent limitations of RNNs in modeling long-range dependencies and facilitate selective contextual modeling across frequency dimensions. When synthesized, these elements enhance the model’s capability to capture intricate timbral evolutions and rhythmic patterns, thereby providing a more precise representation of the distinctive attributes of each instrument in multi-instrument performance contexts.

The terminal output *H*′ functions as the input to ensuing instrument-specific projection modules, thereby offering a unified and semantically enriched representation for each designated instrument.

#### Multi-channel feature projection.

Upon acquiring the comprehensive feature representation $H^{\prime }\in {\mathbb{R}}^{d^{\prime \prime }\times T^{\prime }}$ that assimilates contextual information across temporal and spectral domains, the model subsequently projects this representation into *N* high-dimensional embeddings. Each embedding is aligned with a distinct category of Chinese traditional instruments. To facilitate this, an autonomous linear projection head is designated for each instrument, enabling the simultaneous transformation of the shared representation.


$$E_{i}={\text{Linear}}_{i}(H^{\prime })\in {\mathbb{R}}^{d_{e}\times T^{\prime }},\;i=1,\ldots ,N$$ (11)

The resulting output embeddings are concatenated along the channel dimension to form a three-dimensional tensor:


$$E=[E_{1},\ldots ,E_{N}]\in {\mathbb{R}}^{N\times d_{e}\times T^{\prime }}$$ (12)

The tensor *E* represents the final output of the separation network, which captures dynamic structured representations of the target instrument classes *N* in a unified time-frequency embedding space. This representation retains localized spectral patterns aligned with the original frequency distribution, while integrating contextual semantics across temporal and frequency dimensions. It serves as a rich and discriminative multichannel input for the subsequent classification network.

### Classification network

Following the execution of the separation process, which disentangles time-frequency features and constructs models for various instrument channels, the system progresses to the subsequent phase: a multi-label classification task. The objective of the classification network is to ascertain the presence or absence of each instrument within the input mixture, generating a multilabel prediction vector $\hat{y}\in [0,1{]}^{N}$ where *N* denotes the number of target instrument classes.

To address the diverse representational features of various instruments and the distinct requirements of practical applications, we employ a modular and extensible architecture for the classification network. The general pipeline consists of three stages: (1) *feature extraction*, which processes the input spectrogram through convolutional or attention-based modules to capture local and global patterns; (2) *temporal aggregation*, which summarizes the time-varying features into a fixed-dimensional representation; and (3) *classification head*, which maps the aggregated features to class probabilities through fully connected layers with sigmoid activation:


$${\hat{y}}_{i}=\sigma (f_{\text{cls}}(f_{\text{agg}}(f_{\text{ext}}(X)))),\;i=1,\ldots ,N$$ (13)

Here, *f*_ext_, *f*_agg_, and *f*_cls_ denote the feature extraction, aggregation, and classification functions, respectively, and σ(·) is the sigmoid activation. The output ${\hat{y}}_{i}\in (0,1)$ indicates the predicted probability that the *i*-th instrument is present in the input.

To assess the system’s generalizability across various modeling assumptions, we conducted experiments utilizing a range of classifier architectures with different levels of complexity. These included: (1) Multi-Layer Perceptrons (MLPs) with stacked fully connected layers for direct feature-to-class mapping; (2) Convolutional Neural Networks (CNNs) with hierarchical convolutional blocks to capture multi-scale spatial-spectral patterns; (3) Convolutional Recurrent Neural Networks (CRNNs) combining convolutional feature extraction with bidirectional GRU layers for sequential dependency modeling; and (4) Vision Transformer (ViT) based models that partition the spectrogram into patches and leverage multi-head self-attention for global context modeling. The detailed architecture specifications are provided in the Experiments section.

In our training approach, we utilize the binary cross-entropy (BCE) loss function to optimize the multi-label classification objective. To address the data imbalance among instrument classes, particularly where certain instruments are underrepresented, we implement class reweighting strategies and optionally apply the Focal Loss to enhance the robustness of the model. Serving as the decision-making component at the backend of the system, the classification network efficiently discriminates and categorizes features extracted by the separation module, while offering scalability and adaptability for a broad spectrum of Chinese traditional music recognition tasks.

### Model optimization

To effectively train both the separation and classification networks, we design task-specific loss functions and a joint optimization strategy that enable the model to simultaneously achieve strong feature disentanglement and accurate classification performance. The entire system is trained from start to finish, where the separation and classification modules share a common training objective while maintaining independent gradient flows and update paths.

#### Separation network optimization.

The objective of optimizing the separation network is to enhance the disentanglement capability between instrument-specific feature channels. Each channel is expected to focus on features relevant to its corresponding instrument class, minimizing redundant or overlapping information. Since the separation module does not perform waveform reconstruction, conventional waveform or spectrogram reconstruction losses are not applicable in this context. Instead, we design a feature separability–driven loss function based on the following principles.

First, for each target instrument class, we expect the corresponding feature channel to exhibit higher activation energy when the instrument is present compared to when it is absent. To encourage this, we define a global energy contrastive loss as:


$${ℒ}_{\text{sep}}=\frac{1}{N}\sum_{i=1}^{N}{\left({𝔼}_{x\in {𝒫}_{i}}[||E_{i}|{|}_{1}]-{𝔼}_{x\in {𝒩}_{i}}[||E_{i}|{|}_{1}]\right)}^{2}$$ (14)

Here, ${𝒫}_{i}$ and ${𝒩}_{i}$ denote the sets of positive and negative samples for the *i*-th instrument class, respectively, and *E*_*i*_ represents the characteristic output of the corresponding channel. This loss encourages the model to activate the correct channel when the target instrument is present while suppressing spurious activations for irrelevant classes, thereby improving the discriminability of the separated features.

Additionally, to further reinforce the structural independence between channels, we introduce an inter-channel feature correlation penalty:


$${ℒ}_{\text{orth}}=\sum_{i\neq j}||\text{Corr}(E_{i},E_{j})|{|}_{F}^{2}$$ (15)

where Corr(·,·) denotes the correlation between the feature channels in the temporal dimension, and $||\cdot |{|}_{F}$ is the Frobenius norm. This term encourages the network to learn orthogonalized feature representations across different instrument channels, further improving the quality of separation.

The final loss for the separation network is a weighted combination of the above components:


$${ℒ}_{\text{separation}}=\lambda_{1}{ℒ}_{\text{sep}}+\lambda_{2}{ℒ}_{\text{orth}}$$ (16)

where $\lambda_{1}$ and $\lambda_{2}$ are balancing hyperparameters, empirically set to 1 and 0.5, respectively, in our experiments.

#### Classification network optimization.

The classification network addresses a multilabel binary classification task and adopts the Binary Cross Entropy (BCE) loss as the primary objective to measure the discrepancy between predicted probabilities and ground-truth labels for each class. The standard BCE loss is defined as


$${ℒ}_{\text{cls}}=-\frac{1}{N}\sum_{i=1}^{N}\left(y_{i}\log({\hat{y}}_{i})+(1-y_{i})\log(1-{\hat{y}}_{i})\right)$$ (17)

where $y_{i}\in \{0,1\}$ is the binary label for the *i*-th instrument class, and ${\hat{y}}_{i}\in (0,1)$ is the predicted probability. Given the highly imbalanced distribution of instrument categories in real-world data, we extend the loss of BCE with class-specific weights $\alpha_{i}$ to improve the model’s sensitivity to underrepresented classes:


$${ℒ}_{\text{w-cls}}=-\frac{1}{N}\sum_{i=1}^{N}\alpha_{i}\left(y_{i}\log({\hat{y}}_{i})+(1-y_{i})\log(1-{\hat{y}}_{i})\right)$$ (18)

The weights $\alpha_{i}$ are calculated as the inverse of the class frequencies in the training set, normalized to ensure stability during optimization.

For data subsets with significant class co-occurrence and label overlap, we further experiment with Focal Loss as a replacement for standard BCE. This loss reduces the influence of easy-to-classify samples and directs the model’s focus toward harder instances:


$${ℒ}_{\text{focal}}=-\sum_{i=1}^{N}\alpha_{i}(1-{\hat{y}}_{i}{)}^{\gamma }\log({\hat{y}}_{i})$$ (19)

Here, γ is a focus parameter, set to 2 in our experiments. This loss function demonstrates notable improvements in recognizing overlapping instruments during comparative evaluations.

## Results

### Dataset

To evaluate the effectiveness of the proposed “separation-then-classification” strategy for the recognition of Chinese traditional instruments, we constructed a dedicated audio data set designed for the separation and recognition of lead instruments in mixed multi-source scenarios. The dataset includes four representative Chinese instruments, guzheng, pipa, xiao, and dizi, all commonly used as lead parts in real-world musical compositions and performances. These instruments are characterized by distinctive timbral qualities and stylistic characteristics. All audio samples were collected from original musical projects led by the authors, ensuring high artistic quality, stylistic diversity, and authentic performance expression. This avoids the common problems of clipping artifacts and repetitive content often found in commercial copyright databases.

Each sample in the dataset contains three aligned audio tracks: (1) a *mix track* consisting of both the target instrument and its accompaniment, serving as the recognition input; (2) an *accompaniment track* containing the mixture with the lead instrument removed; and (3) a *solo instrument track* with only the clean signal from the target instrument (guzheng, pipa, xiao or dizi). This tri-track structure facilitates precise target-source localization in supervised learning settings and supports downstream recognition and analytical tasks based on source separation.

**Fig 2:**
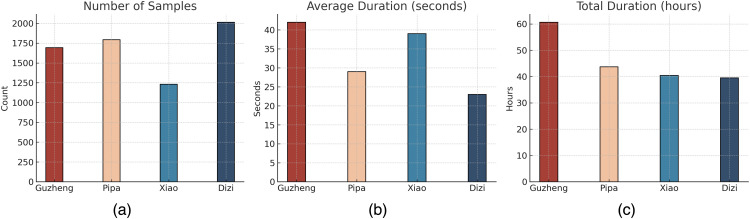
Statistical overview of the dataset. A: Number of samples per instrument. B: Average duration per audio clip (seconds). C: Total duration per instrument (hours).

In total, the dataset contains more than 184 hours and 13 minutes of audio recordings, comprising 12,192 files and 6,731 labeled samples, making it one of the largest publicly available resources in the domain of Chinese traditional instrument data. Detailed statistics are shown in Table 1, and a visual overview of the dataset distribution is presented in Fig 2.

**Table 1:** Statistics of the Chinese traditional instrument dataset.

Instrument	Samples	Files	Total	Avg	Duration Ratio
Guzheng	1,694	5,082	60h 37m 05s	42 sec	32.9%
Pipa	1,793	5,379	43h 42m 19s	29 sec	23.7%
Xiao	1,231	3,692	40h 24m 17s	39 sec	21.9%
Dizi	2,013	6,039	39h 29m 55s	23 sec	21.4%
**Total**	**6,731**	**12,192**	**184h 13m 37s**	–	**100%**

These statistics highlight several notable characteristics. The dizi has the highest number of samples (2,013), but the shortest average duration (23 seconds), indicating that it often appears in short melodic phrases. In contrast, the guzheng has fewer samples (1,694) but the longest total duration (more than 60 hours), with each segment averaging 42 seconds, reflecting its sustained use in structural sections. The xiao also exhibits a relatively long average duration (39 seconds), likely due to its role in expressing smooth, continuous melodic lines. The pipa shows a balanced distribution between the sample count and the average duration.

In terms of stylistic diversity, the dataset includes both traditional solo instrumental styles and modern expressions of Chinese music, such as film soundtracks, crossover pop, and electro-acoustic fusions. This multidimensional stylistic coverage enables models to be trained not only in clean conditions but also in more complex and realistic mixture contexts, thereby enhancing generalization and applicability.

In general, the dataset demonstrates a systematic and application-oriented design in terms of sample structure, duration distribution, stylistic coverage, and multitrack organization. Fills a significant gap in the field of lead instrument recognition for Chinese traditional music and serves as a solid foundation for source separation and classification experiments in this study.

### Training setup

All experiments were conducted on four NVIDIA RTX 4090 GPUs, running Ubuntu 22.04. The deep learning framework used was PyTorch 2.0 with CUDA 12.1, and mixed precision training was enabled to improve computational efficiency and memory utilization. To ensure reproducibility, all training runs were performed with fixed random seeds.

The separation network was trained independently for 100 epochs, with a batch size of 16. The optimizer used was Adam with an initial learning rate of 1 × 10^−4^, and a cosine annealing scheduler was applied to dynamically adjust the learning rate over the course of training.

The classification networks were trained independently for each model, with the same maximum number of 100 epochs. To prevent overfitting, an early stopping mechanism was employed: If no performance improvement was observed in the validation set for 10 consecutive epochs, training was terminated early. The optimizer for classification was also Adam, initialized with a learning rate of 5 × 10^−4^. A ReduceLROnPlateau scheduler was used to automatically reduce the learning rate when the validation performance plateaued.

### Experimental design

First, we compare the classification method proposed in this study with three representative approaches from previous work [29,30,34]. All models were trained in the experimental configurations described in the Training setup subsection.

To evaluate the effectiveness of the proposed separation-based classification method for traditional Chinese instruments in practical tasks, a controlled experimental design was adopted. Specifically, we evaluated the classification performance of models with and without the integration of the separation network. The classification task is formulated as a four-class problem that targets four commonly used Chinese instruments: guzheng, dizi, pipa, and xiao. All experiments were carried out under the same dataset partitioning, training epochs, and hyperparameter settings. The main focus was to compare the impact of the two input conditions on the accuracy of the final recognition and to analyze the role of the separation module in the extraction and classification of features of the instrument.

In the baseline configuration without the separation network, the classification model directly takes the mixed audio track, which contains both the target instrument and accompaniment, as input, learning to distinguish instrument types in complex acoustic backgrounds. In contrast, in the separation network configuration, the input audio is first processed by the trained acoustic separation module to extract a relatively clean solo track of the target instrument, which is then fed into the classifier. The structure of the classification model remains identical across both conditions, ensuring that any observed performance differences can be attributed solely to the acoustic structure of the input. This design guarantees the scientific validity and fairness of the comparison.

To cover models of varying complexity and architectural types, we selected four representative deep learning architectures for comparison, ranging from lightweight to high-capacity structures. The selected architectures include MLP, CNN, CRNN, and Transformer, all of which have demonstrated effectiveness in speech, music, and acoustic recognition tasks. These models are suitable for evaluating the generalizability and performance gains introduced by the separation module under different network configurations.

For each model, two experiments were conducted using: (1) the original mixture input and (2) the separated audio input. The final classification performance was evaluated using four metrics: precision, macro precision, macro recall, and macro F1 score. Detailed results and analysis are presented in subsequent sections. This experimental design aims to systematically reveal the mechanism by which acoustic feature separation improves classification performance in complex audio recognition scenarios.

The detailed architecture specifications of the four classification models are summarized in Table 2. The MLP employs a three-layer fully connected architecture with progressively decreasing hidden dimensions (1024 → 512 → 128) for hierarchical feature abstraction. The CNN utilizes four convolutional blocks with increasing channel depths to extract multi-scale spatial-spectral patterns. The CRNN combines convolutional feature extraction with stacked bidirectional GRU layers for sequential dependency modeling. The Transformer follows the Vision Transformer (ViT) paradigm, partitioning the input spectrogram into non-overlapping patches, projecting them into a latent embedding space, and processing the sequence through multi-head self-attention layers for global context modeling. All models share common components including batch normalization, dropout regularization, and appropriate activation functions.

**Table 2:** Architecture specifications of the classification models.

Model	Feature Extraction	Aggregation	Classifier
MLP	—	Flatten	3×FC (1024-512-128-4)
CNN	4×Conv (64-128-256-512)	Flatten	2×FC (512-128-4)
CRNN	3×Conv + 2×BiGRU	Last state	2×FC (512-128-4)
Transformer	PatchEmbed + 2×Encoder	[CLS]	FC (256−4)

Note: Input: 128-dim Mel-spectrogram (10s, SR = 44.1kHz). Conv: 3×3 kernel, BN, ReLU, MaxPool, Dropout. BiGRU: hidden = 256. Transformer: patch = 16×16, *d* = 256, heads = 4.

In addition to the four proposed architectures, we also include three external baseline methods for comprehensive comparison: (1) RBF-SVM, a traditional machine learning approach using radial basis function kernels on handcrafted features; (2) CNN + SVM, a hybrid method combining CNN-based feature extraction with SVM classification; and (3) ResNet-18, a widely adopted deep residual network pretrained on large-scale audio datasets. These baselines represent different paradigms in audio classification and provide reference points for evaluating the effectiveness of our proposed framework.

### Experimental results and analysis

#### Hyperparameter exploration for frequency band ratio.

Prior to conducting the main classification experiments, we performed a systematic hyperparameter search to determine the optimal frequency band ratio configuration ($\alpha_{1}$, $\alpha_{2}$, $\alpha_{3}$) for the source separation network. Given that the spectral energy of Chinese traditional instruments, including guzheng, pipa, xiao, and dizi, is predominantly concentrated in the mid-to-high frequency range, we constrained the search space for the low-frequency band ratio $\alpha_{1}$ to the range of 15% to 30%. A total of 13 configurations were evaluated using the CNN+ model, with results averaged over 10 independent runs. The detailed results are presented in Table 3.

**Table 3:** Hyperparameter exploration for frequency band ratio configurations using the CNN+ model (mean ± std over 10 runs).

$\alpha_{1}$	$\alpha_{2}$	$\alpha_{3}$	Acc.	Prec.	Rec.	F1
(%)	(%)	(%)	(%)	(%)	(%)	(%)
15.00	40.40	44.60	91.33±1.11	91.83±1.81	92.15±1.68	91.94±1.67
16.25	39.80	43.95	92.81±1.09	93.07±1.81	93.23±1.68	92.83±1.61
17.50	39.21	43.29	93.22±1.08	93.39±1.78	93.72±1.63	93.93±1.59
18.75	38.62	42.63	94.00±1.03	94.16±1.74	94.92±1.61	94.69±1.56
20.00	38.02	41.98	95.06±1.00	94.83±1.71	95.60±1.57	95.07±1.53
21.25	37.43	41.32	95.75±0.98	95.70±1.68	95.94±1.54	95.99±1.51
22.50	36.83	40.67	96.28±0.95	96.24±1.65	96.62±1.51	96.46±1.47
**23.75**	**36.24**	**40.01**	**96.65±0.92**	**96.69±1.62**	**97.06±1.48**	**96.89±1.44**
25.00	35.65	39.35	96.21±0.95	96.23±1.65	96.59±1.51	96.51±1.47
26.25	35.05	38.70	95.65±0.98	95.79±1.68	96.22±1.54	95.83±1.50
27.50	34.46	38.04	95.26±1.02	95.27±1.71	95.55±1.56	95.48±1.53
28.75	33.86	37.39	94.14±1.04	94.60±1.73	94.38±1.61	94.17±1.56
30.00	33.27	36.73	93.78±1.08	93.36±1.77	94.04±1.63	93.24±1.60

As shown in Table 3, the classification performance exhibits a clear inverted-U pattern with respect to the low-frequency band ratio $\alpha_{1}$. When $\alpha_{1}$ is set too low (e.g., 15%), the model allocates excessive capacity to the mid-to-high frequency bands, potentially causing over-segmentation of harmonically related spectral components. Conversely, when $\alpha_{1}$ is set too high (e.g., 30%), the low-frequency band captures spectral regions with minimal instrumental energy, introducing noise into the learned representations. The optimal configuration was identified at $\alpha_{1}=23.75\%$, $\alpha_{2}=36.24\%$, and $\alpha_{3}=40.01\%$, achieving the highest accuracy of 96.65% and F1-score of 96.89%. This configuration was adopted for all subsequent experiments reported in this study.

#### Comparison with baseline models.

To systematically assess the effectiveness of the proposed “separation-then-classification” strategy for Chinese traditional instrument recognition, we compare its performance with several traditional baseline models (RBF-SVM, CNN + SVM, ResNet-18) in identical data partitioning and training configurations. We also compare the results with four deep learning architectures proposed in this study, both with and without the integration of the separation module. All experiments were repeated ten times and the average results along with the standard deviations are reported. The detailed results are shown in Table 4.

**Table 4:** Performance comparison between the proposed method and baseline models on Chinese traditional instrument classification (four-class task, mean ± standard deviation over 10 runs).

Model	Accuracy (%)	Precision (%)	Recall (%)	F1-score (%)
RBF-SVM	46.53 ± 1.85	48.10 ± 2.10	45.20 ± 2.35	46.60 ± 2.00
CNN + SVM	74.31 ± 1.25	75.00 ± 1.40	73.50 ± 1.50	74.20 ± 1.35
ResNet-18	77.63 ± 1.10	78.10 ± 1.20	76.80 ± 1.35	77.40 ± 1.15
MLP+	75.96 ± 1.10	76.09 ± 1.63	75.63 ± 1.16	75.85 ± 1.03
CNN+	96.65 ± 0.92	96.69 ± 1.62	97.06 ± 1.48	96.89 ± 1.44
CRNN+	93.35 ± 0.73	93.98 ± 1.06	93.20 ± 1.40	93.59 ± 1.11
Transformer+	86.68 ± 1.03	85.78 ± 1.52	87.81 ± 1.71	86.78 ± 1.38

Note: The “ + ” symbol indicates that the model incorporates the source separation module during the classification stage, using the extracted solo instrument audio as input. Models without the “ + ” symbol are baseline approaches that directly classify the mixed audio input.

From the perspective of overall performance, the four models proposed in this study (MLP + , CNN + , CRNN + , Transformer+) significantly outperform traditional baselines in all four metrics: accuracy, precision, recall, and F1 score. This validates the substantial advantage of incorporating a source separation mechanism in improving lead instrument recognition. Among them, the CNN+ model achieved the best results, with an accuracy of 96.65% (±0.92) and an F1-score of 96.89% (±1.44), outperforming the traditional CNN + SVM model (accuracy: 74.31% ±1.25) by over 22 percentage points. This indicates that when provided with clean, separation-enhanced input features, the CNN architecture can fully exploit its capacity to distinguish subtle timbral differences among instruments.

The CRNN+ model also demonstrated strong recognition capability, achieving an accuracy of 93.35% (±0.73). By incorporating recurrent layers for temporal modeling, it effectively complements the limited receptive field of pure convolutional structures. This advantage is especially evident for instruments such as dizi and xiao, which are characterized by rich duration variations and prominent legato features.

In comparison, the Transformer+ model shows potential in modeling long-term dependencies, reaching an F1-score of 86.78% (±1.38). Although its overall performance is slightly lower than that of CRNN + , it achieved a recall of 87.81% (±1.71), indicating a strong ability to detect target instruments. This suggests that the model is well-suited for processing acoustic inputs with strong contextual continuity.

Despite its relatively simple architecture, the MLP+ model still achieved an accuracy of 75.96% (±1.10) under the enhanced input condition, significantly outperforming its traditional counterpart RBF-SVM (46.53% ±1.85). This result shows that even shallow network structures can deliver competitive classification performance when provided with high-quality input signals. This finding further highlights the foundational role of acoustic front-end processing in determining the effectiveness of the model.

#### Comparison of models with and without source separation.

**Fig 3:**
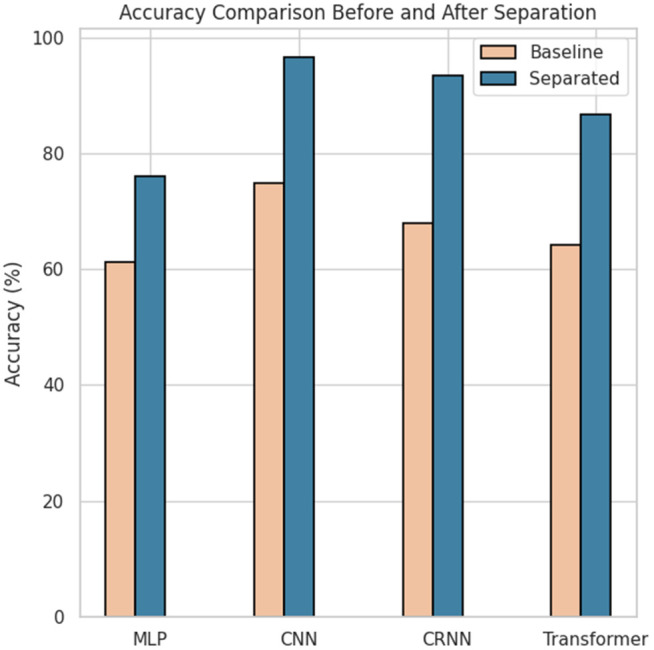
Accuracy comparison before and after incorporating source separation across four classification models. Separation-enhanced inputs (MLP + , CNN + , CRNN + , Transformer+) consistently improve performance over baseline settings.

**Fig 4:**
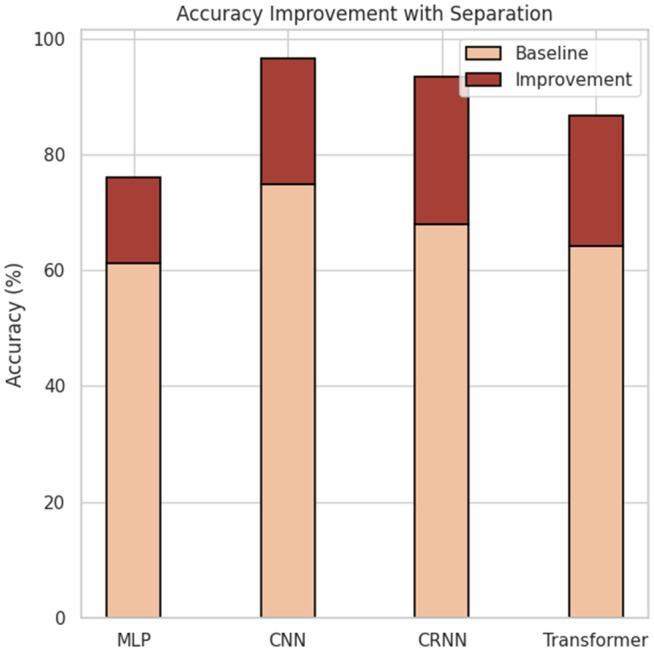
Stacked bar chart showing accuracy improvement achieved by source separation. The darker segment indicates absolute gains over baseline performance for each model.

To further verify the generalizability and effectiveness of the “separation-then-classification” strategy across different model architectures, we conducted a series of comparative experiments on four representative deep learning models: MLP, CNN, CRNN, and Transformer. Specifically, we assessed how the inclusion of a source separation module impacts classification performance. The general results are summarized in Table 5, while the instrument-level performance is detailed in Table 6. Statistical significance analysis is presented in Table 7. The visual comparison of accuracy improvements is illustrated in Fig 3 and Fig 4.

**Table 5:** Performance comparison of different models with and without source separation on Chinese traditional instrument classification (four-class task, mean ± standard deviation over 10 runs).

Model	Accuracy (%)	Precision (%)	Recall (%)	F1-score (%)
MLP	61.30 ± 1.05	61.18 ± 1.71	61.55 ± 1.78	61.36 ± 1.69
CNN	74.81 ± 0.93	74.43 ± 1.55	74.61 ± 1.51	74.51 ± 1.19
CRNN	68.01 ± 1.00	67.83 ± 1.10	68.67 ± 1.07	68.24 ± 0.80
Transformer	64.11 ± 0.91	63.64 ± 1.62	64.32 ± 1.28	63.97 ± 1.25
MLP+	75.96 ± 1.10	76.09 ± 1.63	75.63 ± 1.16	75.85 ± 1.03
CNN+	96.65 ± 0.92	96.69 ± 1.62	97.06 ± 1.48	96.89 ± 1.44
CRNN+	93.35 ± 0.73	93.98 ± 1.06	93.20 ± 1.40	93.59 ± 1.11
Transformer+	86.68 ± 1.03	85.78 ± 1.52	87.81 ± 1.71	86.78 ± 1.38

Note: The “ + ” symbol indicates that the model incorporates a trained source separation network during the input stage, using the extracted solo instrument audio from the mixture track as input to the classifier. Models without the “ + ” symbol use the original mixed audio containing both lead and accompaniment.

**Table 6:** Per-class accuracy comparison of different models on Chinese traditional instrument recognition (mean ± standard deviation over 10 runs).

Model	Guzheng (%)	Dizi (%)	Pipa (%)	Xiao (%)
MLP	60.73 ± 2.94	61.96 ± 1.66	61.55 ± 2.63	60.98 ± 2.56
CNN	75.18 ± 2.00	75.01 ± 2.54	75.10 ± 1.94	73.94 ± 2.75
CRNN	66.44 ± 2.74	68.31 ± 3.55	69.44 ± 2.91	67.86 ± 3.36
Transformer	63.08 ± 2.79	63.82 ± 1.83	64.86 ± 2.41	64.68 ± 1.56
MLP+	77.05 ± 2.13	76.46 ± 2.59	74.84 ± 2.57	75.52 ± 2.68
CNN+	**97.53 ± 2.58**	**95.85 ± 2.90**	**97.36 ± 1.83**	**95.81 ± 2.68**
CRNN+	92.91 ± 2.51	93.35 ± 2.39	94.26 ± 2.45	92.89 ± 2.89
Transformer+	85.88 ± 1.96	87.72 ± 2.93	85.73 ± 2.22	87.39 ± 1.69

Note: The table presents classification accuracy for four target instruments (guzheng, dizi, pipa, xiao) across different models. Models with the “ + ” symbol incorporate a source separation module during the classification stage, using the extracted solo audio of the target instrument. Models without the “ + ” symbol classify directly from the original mixture input.

**Table 7:** Statistical significance analysis of classification improvements with source separation (paired-sample t-test results).

Model	Paired t-stat	P-value	Significance	Cohen’s d	Effect Size
MLP	36.1050	4.748e-11	***	12.9267	Very Large
CNN	38.3573	2.763e-11	***	22.4825	Very Large
CRNN	51.0269	2.141e-12	***	27.5070	Very Large
Transformer	47.4247	4.128e-12	***	21.9930	Very Large

Note: This table reports the statistical results of accuracy improvements under the “separation-then-classification” strategy for four models. Paired-sample t-tests were used to analyze the performance differences between mixed audio input and separation-enhanced input. All reported p-values are less than 0.001 (denoted as “***”), indicating highly significant improvements.

In terms of overall classification performance, all models exhibited substantial performance improvements after incorporating the separation module. For example, the accuracy of the MLP model increased from 61.30% to 75.96%, CNN from 74.81% to 96.65%, CRNN from 68.01% to 93.35%, and Transformer from 64.11% to 86.68%. Similar trends were observed in other evaluation metrics, including precision, recall, and F1 score. These results indicate that the separation module not only enhances the discriminative capacity of each model, but also improves the coverage and robustness of the system across different instrument categories. Among all models, CNN+ achieved the highest performance, with an F1 score of 96.89%, approaching a near-perfect classification. This suggests that convolutional architectures are highly compatible with the timbral features derived from the separation module and are effective in learning from enhanced acoustic inputs.

From a per-category perspective (Table 6), the separation module produced positive impacts across all four classes of instruments. The most notable improvements were observed for Dizi and Xiao. For example, the accuracy of the dizi classification using the CNN model increased from 75.01% (without separation) to 95.85% (with separation), while xiao improved from 73.94% to 95.81%. These gains can likely be attributed to the fact that the timbral features of dizi and xiao are more susceptible to being masked by accompaniment in the mix audio. The separation module successfully extracted the dominant spectral structures of these instruments, enhancing the model’s ability to identify their unique timbral signatures. Although the improvements for Guzheng and Pipa were less pronounced, the performance gains remained consistent across all models, suggesting the broad applicability of the proposed strategy to diverse instrument timbres and playing techniques.

The results of the paired sample test *t* in Table 7 further confirm the statistical reliability of these observations. In all four models, the performance improvements resulting from the separation module reached a high level of statistical significance (*p* < 0.001). Furthermore, the calculated Cohen effect sizes *d* all exceeded 12, classified as “Very Large”, indicating that the observed enhancements are not only statistically significant but also practically substantial. Notably, CRNN and Transformer models achieved particularly high effect sizes of 27.51 and 21.99, respectively, underscoring the heightened sensitivity of temporal modeling architectures to the enhanced acoustic features produced by source separation. These structures are more adept at capturing the continuity and dynamic variations inherent in the timbral evolution of traditional instruments.

In general, the “separation-then-classification strategy” proposed in this study consistently yielded performance improvements across different model architectures and instrument categories. These improvements were validated through both quantitative metrics and statistical analysis. This approach not only enhances the capability of classification models in identifying lead instruments under complex mixture conditions but also offers a feasible pathway for downstream tasks such as intelligent music analysis and automatic annotation in real-world multi-source musical environments.

To investigate the necessity of deep classification networks and evaluate how much discriminative information is inherently contained in the separation module output, we conducted an ablation study comparing classifiers of varying complexity. Specifically, we tested three types of classifier configurations ranging from a minimal linear layer to full-scale deep networks. The results are presented in Table 8.

**Table 8:** Ablation study on classifier complexity using separation-enhanced input (mean ± std over 10 runs).

Classifier	Acc. (%)	Prec. (%)	Rec. (%)	F1 (%)
Linear (GAP + FC)	26.83 ± 2.15	27.41 ± 2.68	26.52 ± 2.43	26.94 ± 2.31
MLP-Lite (1×FC-128)	38.25 ± 1.87	39.12 ± 2.14	37.89 ± 2.06	38.47 ± 1.95
MLP (3×FC)	75.96 ± 1.10	76.09 ± 1.63	75.63 ± 1.16	75.85 ± 1.03
CNN-Lite (2×Conv)	77.42 ± 1.05	77.87 ± 1.48	77.15 ± 1.32	77.49 ± 1.21
CNN+	96.65 ± 0.92	96.69 ± 1.62	97.06 ± 1.48	96.89 ± 1.44

The results reveal a clear positive correlation between classifier complexity and classification performance. The minimal Linear classifier achieves only 26.83% accuracy, which is close to random guessing for a four-class problem (25%), indicating that the raw separation output does not directly encode sufficient discriminative information without further feature transformation. Adding a single hidden layer (MLP-Lite) improves accuracy to 38.25%, while the full three-layer MLP reaches 75.96%, demonstrating that hierarchical feature abstraction is essential for extracting meaningful patterns. Introducing convolutional operations yields further improvements: CNN-Lite (2 layers) achieves 77.42%, and CNN+ reaches 96.65%. These findings confirm that the classification network plays a critical role in the proposed framework, and the performance gains cannot be solely attributed to the separation module.

## Discussion

### The role of separation networks in traditional instrument classification

Experimental results indicate that the incorporation of acoustic feature separation networks significantly improves the performance of all models in the classification task of traditional Chinese musical instruments. In particular, consistent and substantial gains were observed in multiple evaluation metrics, including precision, macro precision, macro recall, and macro F1 score. This improvement is not limited to baseline models, but is also evident in more complex architectures such as convolutional neural networks (CNNs), residual networks, and transformers. In general, the introduction of separation networks effectively enhances the quality of the input features for classification models and serves as a key factor in achieving performance gains.

From the perspective of acoustic modeling, the primary function of separation networks is to reduce background interference and improve signal purity. In real-world recording and performance scenarios, traditional instruments are often embedded in complex acoustic environments. Elements such as accompaniment, percussion, synthesized tracks, and environmental reverberation can significantly alter the spectral structure and dynamic characteristics of the original instrument sounds. In particular, in the data sets used in this study, each mixed track typically contains a dominant target instrument along with various background sources. When classification models are trained directly on such mixed signals, the extracted features are often contaminated by nontarget sources, impairing the model’s ability to delineate category boundaries and, in some cases, leading to misclassification.

With the integration of separation networks, the input to the model is transformed from a mixed signal into a relatively “clean” solo track of the target instrument. This process achieves a structural reconstruction of the acoustic signal in the physical sense and provides a more distinct basis for discrimination at the learning level. For instance, the glissando and finger-tremolo techniques of the guzheng are prone to being masked by percussion or background harmonics in mixed audio. After separation, these features, characterized by continuous frequency slides, become more prominent in the spectrogram, thereby facilitating the convolutional kernels to capture essential local structures. Similarly, the vibrato and weak-onset breath patterns of instruments such as the dizi and xiao, which are otherwise susceptible to suppression by compression algorithms or reverberation, exhibit enhanced timbral granularity and dynamic detail after reconstruction. This allows the model to more accurately identify the expressive characteristics of these instrument categories.

Furthermore, from the perspective of feature representation in machine learning, the separation network functions as a form of acoustic preprocessing that enhances the discriminability of target categories in the feature space. Its output signals not only approximate the physical characteristics of real solo instrument performances but also exhibit more stable time-frequency statistical patterns. This leads to faster convergence and more stable parameter updates during the training of downstream classification models. Such feature purification is particularly beneficial in scenarios where generalization capability must be maintained despite limited training samples, and it provides methodological support for the development of low-resource classification systems for traditional music.

In addition, the separation network demonstrates a form of implicit attention guidance. In mixed audio tracks, the target instrument may be acoustically masked by other sources, resulting in underrepresented features in the input representation. During training, the separation network learns to emphasize prominent regions associated with the target instrument, effectively introducing a soft alignment mechanism at the semantic level. This mechanism facilitates a more robust mapping between the input signal and the target label, thus improving both the accuracy and robustness of the model.

It is important to note that while the separation network does not participate directly in the classification task, it plays a decisive role in overall system performance as a front-end feature extraction module. This pre-enhancement strategy differs from conventional methods that apply attention mechanisms or regularization constraints directly in the feature space. Instead, it achieves input purification at the data level, offering advantages such as model independence, structural compatibility, and generalizability of the task. In this study, all model architectures showed varying degrees of performance improvement after incorporating the separation network, which supports its effectiveness as a general-purpose enhancement mechanism.

In summary, the functional role of the separation network in the classification of Chinese traditional musical instruments can be delineated at three levels: (1) audio purification at the signal level, effectively removing interference components; (2) target enhancement at the feature level, improving class separability and model convergence; and (3) structural compatibility at the system level, allowing applicability across different model architectures and acoustic input conditions. Through the combined effect of these three mechanisms, the separation network demonstrates strong performance enhancing capabilities in the classification tasks designed in this study, offering a replicable and scalable preprocessing paradigm for intelligent acoustic analysis in complex traditional music environments.

### Differential model responses to separation-based enhancement

Although all models achieved varying degrees of performance improvement after the integration of the source separation module, notable stratified differences in responsiveness were observed across model architectures, particularly in terms of changes in classification accuracy and F1 score. In general, models with lower capacity and weaker feature extraction capabilities exhibited a greater dependence on separation-based enhancement. In contrast, models with more complex structures, which demonstrated a certain level of robustness under original mixed-input conditions, were still able to benefit from the optimized acoustic input provided by the separation network.

For basic neural network structures such as the multilayer perceptron (MLP), the classification accuracy under mixed input conditions was only 61.30%, significantly lower than that of other models. The corresponding F1 score was 61.36%, indicating the difficulty of the model in effectively identifying the lead instrument within complex acoustic backgrounds. However, after incorporating the separation network, the precision increased to 75.96% and the F1 score increased to 75.85%, marking an improvement of more than 14 percentage points. This change suggests that the separation module plays a dual role in the MLP: purifying input features and reinforcing category boundaries. These functions help alleviate the performance bottleneck caused by the lack of spatial modeling capability of the MLP. Since MLPs cannot perceive local contextual structures, their classification performance is highly dependent on the clarity and semantic boundary of the input signals. The cleaner and more structured solo audio provided by the separation module enables the model to significantly enhance its discriminative power.

In models of moderate complexity, such as the convolutional recurrent neural network (CRNN), the response to separation-based enhancement is even more pronounced. The precision of the CRNN improved from 68.01% to 93.35%, while the F1 score increased substantially from 68.24% to 93.59%, reflecting a performance gain of more than 25 percentage points. This represents a leap from a moderate-performing model to one with high performance. These results suggest that the CRNN, which combines convolutional feature extraction with temporal modeling, can more effectively activate its acoustic dynamic modeling capacity when provided with input audio that has a clear time-frequency structure. This is particularly beneficial for instruments such as the dizi and xiao, which are characterized by sustained tones and ornamental articulations. In this case, the separation module not only improves the quality of local signal features but also enhances the learnability of global temporal dependencies, resulting in a performance amplification effect.

In high complexity architectures such as CNN+ and Transformer+ models, the baseline performance under mixed input conditions was already relatively high (e.g., 74.81% accuracy for CNN and 64.11% for Transformer). However, after incorporating the separation module, significant improvements were still observed. The CNN+ model achieved an accuracy of 96.65% and an F1 score of 96.89%, while the Transformer+ model stabilized at 86.68% accuracy and 86.78% F1 score. These performance gains suggest that separation-based enhancement is not only beneficial for models with limited capacity, but also provides additional modeling space for complex architectures with strong learning capabilities. In particular, although the performance gain of the Transformer (an increase of 22.57 percentage points in accuracy) was not as large as that of the CRNN, the cleaner input enabled its attention mechanism to focus more precisely on the target signal regions. This effectively mitigated issues such as attention dispersion and feature occlusion under mixed inputs, validating the robust generalization of the Transformer in post-separation conditions.

Moreover, the CNN+ model, representing moderately complex yet well-optimized architectures, already achieved 74.81% accuracy and 74.51% F1 score with raw inputs. After incorporating the separation module, its precision increased to 96.65% and the F1 score to 96.89%, approaching optimal performance. This indicates that CNN-based models are highly adaptive to separation-enhanced inputs. Convolutional kernels are particularly effective at capturing timbral and pitch-related features, demonstrating a strong responsiveness to purified input features, as well as improved training stability and convergence efficiency.

Together, the performance gains from the separation network in different model structures can be categorized into three types. For basic models such as MLP, the main function is to compensate for the limited modeling capacity. For moderately complex models such as CRNN and CNN, the Separation Module acts to amplify the learning potential unlocked by higher quality data. For advanced models like Transformer, while the module does not alter the inherent modeling capacity, it significantly improves semantic focus, thereby enhancing the discriminative power of deep structural layers. This stratified pattern of responsiveness highlights the generality and configurability of the separation strategy as a front-end processing module. In future applications of multi-source instrument recognition systems, the use of separation modules can be flexibly adjusted based on model size, task complexity, and computational constraints, in order to optimally balance recognition performance and resource efficiency.

### Applicability and limitations of the method

Based on the experimental results and the performance across different models, the enhancement mechanism using acoustic feature separation demonstrates a high degree of generalizability and adaptability in the task of classifying Chinese traditional musical instruments. Whether applied to lightweight models, convolutional neural networks, or deeper architectures such as residual networks and Transformers, this method consistently yields stable and significant performance improvements. The experiments show that the introduction of the separation network leads to simultaneous gains in accuracy, precision, recall, and F1 score across all models. This indicates that acoustic separation not only improves the discriminative quality of the input signals but also enhances the models’ ability to focus on relevant features. As the preprocessing mechanism is independent of any specific model architecture, it possesses strong transferability and universality, making it suitable for broader acoustic classification tasks, particularly in scenarios requiring the extraction of target signals from noisy or multisource environments.

This is especially relevant in the real-world context of traditional and ethnic music, where multi-source recordings, complex orchestration, and low signal-to-noise ratios are common technical challenges. Traditional instruments’ performances often involve sophisticated techniques such as glissandi, ornamental figures, and breath control. These details are highly susceptible to being masked by other sound sources in mixed signals, making it difficult for classification models to capture critical features. The separation-based enhancement mechanism proposed in this study effectively removes background interference and reconstructs the solo track of the target instrument, thereby providing a clear and structured input representation for subsequent modeling. As a result, the robustness of the model under complex conditions is significantly improved. This approach offers a foundational technical solution for the development of large-scale, stylistically diverse, and structurally complex intelligent analysis systems for traditional music.

However, this method still presents certain limitations, primarily in terms of its application scope and implementation cost. First, the quality of acoustic separation has a direct and significant impact on the final classification performance. If the separation network produces distortions in timbre, a rhythm misalignment, or incomplete extraction of the target signal, it can adversely affect the decision-making of the downstream model and even lead to misclassification. Second, the current approach is designed for a single-instrument, four-class classification task, and lacks direct adaptability to more complex scenarios involving multilabel classification or simultaneous multi-instrument inputs. Moreover, training a high-quality separation network requires well-annotated data, particularly isolated solo tracks to serve as supervision signals, which are often difficult to obtain in practical data collection processes.

Future research could explore the integration of weakly supervised learning, pseudolabel generation, or end-to-end joint optimization strategies to reduce dependency on finely labeled datasets. Furthermore, this method could be extended to support multimodal inputs, multitask learning, and automated acoustic recognition systems under real-world performance conditions.

## Conclusion

The acoustic enhancement framework proposed in this study—based on a ’separation-then-classification’ strategy—demonstrates significant advantages in the multi-source recognition task of traditional Chinese musical instruments. Across various model architectures, including MLP, CNN, CRNN, and Transformer, the application of this method consistently resulted in substantial performance improvements, significantly outperforming comparable models that did not utilize source separation. This strategy not only enhances the discriminability of input signals, but also effectively improves the models’ ability to focus on relevant features and perform structural modeling. It exhibits a particularly notable advantage in low-cost, high-gain compensation for shallow and moderately complex models, while further activating fine-grained feature-modeling capabilities in high-complexity models.

Compared to conventional approaches that perform direct classification, the proposed system achieved notable improvements in multiple metrics, including accuracy, precision, recall, and the F1 score, with the highest accuracy reaching 97.53%. The method is structurally agnostic and highly generalizable, allowing it to be adapted to different model architectures and practical task conditions. Although the current implementation relies on solo-instrument-supervised data to train the separation module, future work may introduce weak supervision or pseudo-labeling strategies to further expand its applicability.

This study provides a systematic solution for instrument recognition in multi-source recording environments of traditional music, with strong potential for real-world applications. Possible extensions include intelligent assisted teaching in educational contexts, digital archive organization, and real-time analysis systems for live performance scenarios.
